# Comparison of Management Strategies and Associated Complications With Slipped Capital Femoral Epiphysis

**DOI:** 10.7759/cureus.92349

**Published:** 2025-09-15

**Authors:** Sneha Thaiparambil, Akanksha Ahuja, Umme Kalsoom, Bhoomi Bagadia, Arun Nair

**Affiliations:** 1 Maternal-Fetal Medicine, Saint Peter's University Hospital, New Brunswick, USA; 2 Medicine, St. George's University, True Blue, GRD; 3 Medicine, Fatima Memorial Hospital College of Medicine and Dentistry, Lahore, PAK; 4 Medicine, Jawaharlal Nehru Medical College, Karnataka Lingayat Education (KLU) Academy of Higher Education and Research (KAHER), Belagavi, IND; 5 Pediatrics, Saint Peter's University Hospital, New Brunswick, USA

**Keywords:** dunn procedure, obesity, pediatric orthopedic surgery, slipped capital femoral epiphysis, surgical management

## Abstract

With the rising incidence of slipped capital femoral epiphysis (SCFE), a thorough understanding of its complications and management is crucial. SCFE primarily affects adolescents between the ages of 10 and 16, targeting the epiphysis of the hip. Notably, the condition is most prevalent among overweight individuals and males. Various surgical interventions, such as in situ pinning and open reduction, are available to treat SCFE. Postoperative complications have had multifactorial implications; however, from this study, the modified Dunn procedure appears to have better outcomes pertaining specifically to fewer reoperations and requiring total hip arthroplasty from osteoarthritis. This study compares and contrasts different fixation methods by analyzing outcomes from published studies. Through this literature review, we evaluate each procedure's advantages and disadvantages to recommend the best action for this condition.

## Introduction and background

Slipped capital femoral epiphysis (SCFE) is a developmental hip condition most commonly seen in adolescents [1]. It primarily affects the normal functionality of the hip by damaging the epiphysis, also known as the growth plate [1]. This occurs when the femoral head slips out of alignment with the neck of the femur, causing an irregular fit within the hip socket [1]. Adolescents aged 10-16 are most at risk due to factors such as obesity, puberty, hormonal imbalances, and endocrine issues [2,3]. The annual incidence of SCFE ranges from 10 to 12 cases per 100,000 adolescents, with males being twice as likely to be affected than females [2]. The highest rates are seen among children who are overweight, as well as those of the African American or Pacific Islander population [2,3].

The various presentations of SCFE can cause significant delays in diagnosis, as the symptoms can be minimal. These symptoms range from hip discomfort, limping, and pain with movement or walking [4,5]. This is often mistaken for "growing pains" or attributed to other conditions, especially since the pain can be vague and may radiate to the groin or knee [4,5]. In other cases, the slippage of the femoral head may be minimal, leading to the diagnosis being missed or delayed in discovery [4,5]. The absence of certain physical findings makes diagnosis challenging, as it relies on assessing the range of motion, particularly internal rotation, flexion, abduction, and external rotation [4,5]. The key techniques used to confirm a SCFE diagnosis begin with an X-ray, followed by advanced imaging such as MRI or CT scans when X-ray results are inconclusive [4,5]. Imaging can also reveal complications such as avascular necrosis (AVN), musculoskeletal deformities, and early-onset arthritis [3-5]. The gold standard management of a stable SCFE is surgical stabilization [4,5]. Treatment methods are chosen from in situ fixation, osteotomy, and femoroacetabular impingement (FAI) treatment [4,5]. A single screw fixation is the most common method used to fix the epiphysis, and the dynamic method is especially considered for young patients [4,5].

Traditional three-dimensional trochanteric osteotomies have been controversial with regard to their complexity and lack of certainty in patient outcomes [4]. Management differs for an unstable SCFE, as complications are more prevalent, and assessments have to be deciphered in a timely manner [4,5]. It is expected that postoperative AVN can be prevented in many cases through the treatment while also assessing the intraoperative physeal hemodynamics [4,5]. Open surgeries have begun to be indicated in the treatment of unstable SCFE through either an anterior approach or (modified) Dunn procedure [4,5]. The magnitude of stabilizing SCFE depends on the severity of each case and the associated postoperative complications [4,5].

This literature review aims to raise awareness and understanding of the complications and management of SCFE. As the incidence of SCFE rises alongside increasing adolescent obesity rates, there is a growing need for effective management strategies [2,3]. The healthcare system is also witnessing rising costs related to SCFE treatments and aftercare, which include physical therapy, long-term disability support, assisted living conditions, early arthritis, and specialized orthopedic solutions [3,6].

## Review

Methods

The study design of this paper is a literature review, and the databases used to collect articles included PubMed and Google Scholar. The filters applied in the literature search include free full-text with the language exclusively in English, and the year of publication filter applied was between 2010 and 2025. Articles were collected and reviewed by all team members in this study to assess their relevance to the research objectives of this paper. Meetings were conducted virtually, and after consolidating the studies, key points were extracted. Appropriate research questions for this topic were discussed, and articles were delegated appropriately where relevant after a group consensus. Upon drafting the manuscript, two members of the study conducted a critical review of the paper and made edits as required. With only a few pediatric-specific studies that have been done, we have also included adult data where applicable in order to obtain a better sense of the complications of procedures mentioned in this study.

Pathophysiology of SCFE

SCFE is one of the leading causes of hip pain and disability in the pediatric population [1,3,5]. Because SCFE treatment is complex and its pathogenesis remains incompletely understood, identifying precipitating factors is essential for early diagnosis and optimal management [1,3-5]. Pathogenetic mechanisms either decrease the physis's resistance to shear forces or increase stress across the proximal femoral growth plate [1,3,5]. Well-established predisposing factors include obesity, endocrine disorders, femoral or acetabular retroversion, and coxa profunda [2,3]. Histologically, physeal slippage has been linked to the weakening of the collagen network that normally stabilizes the growth plate [3].

Risk factors for SCFE are often divided into "typical" and "atypical" associations. The hallmark typical factor is obesity, whereas atypical associations encompass endocrinopathies, metabolic syndromes, external insults such as radiation or chemotherapy, and nephropathies [3]. In obesity, leptin resistance leads to chronically elevated leptin levels, which in turn expand the proliferative zone of the physis, a key step in SCFE development [3]. Moreover, leptin has been shown to induce chondrocyte hypertrophy, trigger apoptosis, and disrupt type II and type IV collagen integrity within the growth plate [3].

Atypical SCFE more frequently presents bilaterally, with an average bilateral prevalence reported at under 10% [7]. Among these, renal-associated SCFE typically demonstrates the greatest severity and bilaterality, followed by endocrinopathy-related and radiation-associated slips, which tend to be milder [7]. In a 2022 cohort, Goodwin Davies et al. found that children with glomerular disease were 3.4 times more likely to develop SCFE, while obesity conferred a 10.5-fold increased risk [8]. In end-stage renal osteodystrophy, secondary hyperparathyroidism drives excessive bone resorption and osteomalacia, thereby heightening physeal vulnerability [7]. Interestingly, SCFE can also be noted to develop during periods of rapid height velocity and has been reported in several case studies involving growth hormone (GH) therapy [8,9]. 

Comparison of surgical interventions

SCFE remains a challenging condition in pediatric orthopedics, with no clear consensus on optimal management [4,5]. Classification systems vary, complicating the selection of the most appropriate treatment strategy [3,4]. Delayed diagnosis is common and often results in both short-term and long-term functional impairment [4,5].

Once SCFE is confirmed, surgical intervention aims to halt the progression of the slip, relieve impingement, and prevent the development of osteoarthritis [4,5]. AVN and chondrolysis are among the most serious complications [3-5]. In Italy's national registry, AVN occurred in 0% of stable slips versus 47% of unstable slips [10]. More recent series report AVN rates of 0-3.3% in stable SCFE and up to 23.9% in unstable cases [10].

Percutaneous in situ fixation (stabilizing the epiphysis with pins or screws until physeal closure) is the cornerstone of treatment for both stable and unstable SCFE, regardless of deformity severity [4,5]. Although minimally invasive, this approach carries risks of suboptimal reduction, early osteoarthritis (≈15%), and osteonecrosis (10-40%) [3]. Alternative techniques include compensatory osteotomies and direct head‐neck junction corrections; the choice of procedure hinges on the accurate assessment of physeal stability and remaining growth potential [4,5,10].

Kenanidis et al. found that 71-92% of patients with SCFE undergo surgery: 46 underwent closed reduction with pin or screw fixation, 27 had corrective osteotomies, and others required skeletal traction before definitive fixation. Some cases necessitated multiple procedures, while a minority were managed nonoperatively [6]. Hip osteoarthritis may develop over time, often leading to total hip arthroplasty (THA) [3,6,11]. Indications for THA include AVN, chondrolysis, secondary osteoarthritis, and FAI; the interval from initial SCFE to THA ranges from 7.6 to 27.3 years [6,11].

SCFE severity is graded as mild, moderate, or severe by slip angle [4,5]. Mild slips, defined by minimal head displacement, are usually treated with in situ pinning alone [4,5]. Moderate and severe slips carry higher risks of articular cartilage erosion and often benefit from osteochondroplasty at the head-neck junction in addition to stabilization [4,5]. This procedure removes bony prominences to restore sphericity and reduce osteoarthritis risk [4,5].

Osteochondroplasty may be performed arthroscopically or via a limited anterior arthrotomy; in select cases, bone grafting ("osteoplasty") can augment in situ fixation as a less invasive alternative [12]. Realignment osteotomies at the femoral neck, with surgical hip dislocation for access, allow precise deformity correction and may minimize future complications [5,13].

In a cohort of 188 SCFE patients followed for 4.4-11.2 years, surgical approaches included posterolateral (61), posterior (32), anterolateral (79), anterior (3), Hardinge (11), and direct lateral with trochanteric osteotomy (1). Each approach demonstrated improved functional scores and survival rates over the follow-up period (see Table 1) [6].

**Table 1:** Primary outcomes of the included studies HHS: Harris Hip Score; SF-36: 36-Item Short Form Health Survey; LLD: limb-length discrepancy; HO: heterotopic ossification; OHS: Oxford Hip Score; SCFE: slipped capital femoral epiphysis; OA: osteoarthritis; MHHS: Modified Harris Hip Score; OHS: Oxford Hip Score; PMA: Postel-Merle d'Aubigné; VAS: Visual Analogue Score; EQ-5D: EuroQol 5 Dimension; NA: not answered *Results are given as the type with the percentages in parentheses. **Results are given as percentages with the last follow-up (years) in parentheses. ***Results are given as the evaluated test with the mean pre-op and post-op score in parentheses.

Author	Surgical approach (%)*	Survival from revision rate**	Type of fixation (%)*	Functional outcomes***	Other outcomes/complications
Schoof et al. [11]	Posterior (100)	81% (11.2)	Cemented (42). Hybrid (35.4). Uncemented (22.6)	HHS (47-92.3). SF-36 (improved significantly)	No complications. LLD improved (mean pre-op 13.5 mm; mean post-op 6 mm)
Traina et al. [14]	Anterolateral (96.8). Direct lateral/trochanteric osteotomy (3.2)	92.8-96.8%	Cementless (90.5). Hybrid (9.5)	HHS (48.3-86)	HO in 15 hips. LLD (mean pre-op 12 mm; mean post-op 6 mm)
Larson et al. [15]	NA	87% (10)	NA	HHS (51-85)	-
Nizard et al. [16]	NA	82.1% (10), 72.4% (15)	NA	NA	-
Engesæter et al. [17]	NA	-	NA	NA	No difference in revision rate compared to Legg-Calvé-Perthes
Kenanidis et al. [6]	Posterolateral (52.1). Anterolateral (41). Anterior (2.5)	94.8% (4.4)	Cementless (65). Hybrid (29.9). Cemented (5.1)	OHS	No difference in OHS and revision rates between SCFE and OA patients
Hannouche et al. [18]	NA	90.3% (10)	NA	NA	2/12 aseptic loosening at 10 years
Taheriazam and Saeidinia [19]	Hardinge (100)	-	NA	MHHS (49.6-81.2). OHS (18.1-31.2). PMA (8.2-15.4). VAS (6.7-3.6)	-
Lehmann et al. [20]	NA	-	NA	EQ-5D	Significantly higher reported EQ-5D score for SCFE compared to Perthes and hip dysplasia

A comparison between the two surgical techniques is summarized in Table 2. 

**Table 2:** Comparison of in situ fixation vs. modified Dunn procedure

Author	In situ fixation	Modified Dunn procedure
Arora et al. [21]	A single cannulated screw was placed centrally in the epiphysis with a minimum of three threads across the physis. Postoperative rehabilitation was not described.	Ganz safe surgical dislocation with capital realignment; fixation method not described. Intraoperative assessment of femoral head vascularity was performed to ensure there was no vascular pedicle tension. Postoperative rehabilitation was not described.
Galletta et al. [22]	A single 6.5-mm cannulated screw was used. Postoperatively, patients were non-weight-bearing for six weeks.	Postero-inferior callus formation was excised to release tension over the medial femoral circumflex artery; intraoperatively, a 2-mm probe was inserted in the anterior femoral head perforation to check the epiphyseal perfusion. Patients were non-weight-bearing for three months; active abduction and passive adduction were restricted for 4-6 weeks.
Novais et al. [23]	A single 6.5- or 7.3-mm cannulated screw was used in the center of the epiphysis. Postoperatively, patients were partial weight-bearing with crutches for six weeks.	Vascular control was assessed; there was no description regarding the intraoperative monitoring of vascularity. Postoperatively, patients were non-weight-bearing for six weeks, followed by six weeks of protected weight-bearing with crutches.
Trisolino et al. [24]	Two fully threaded 4.5-mm screws were used. Postoperatively, patients were partial weight-bearing for six weeks.	There was no description regarding the intraoperative monitoring of vascularity. Postoperatively, patients were non-weight-bearing for six weeks, followed by six weeks of protected weight-bearing with crutches.

Clinical outcomes were assessed using several validated instruments: two studies employed the Merle d'Aubigné score (Arora et al. [21] and Galletta et al. [22]), one used the Heyman and Herndon classification (Novais et al. [23]), and another applied the Non-Arthritic Hip score (Trisolino et al. [24]). Across these cohorts, the modified Dunn procedure consistently yielded a higher proportion of good to excellent results compared with in situ fixation.

Because SCFE occurs in the growing skeleton, traditional treatment goals prioritize physeal arrest to prevent slip progression from continued femoral neck growth [3,4]. Recent investigations, however, underscore the value of preserving proximal femoral physeal growth and remodeling to avert sequelae such as FAI and limb-length discrepancies [4,5].

Longitudinal analyses reveal a significant uptick in secondary procedures, treatment-related complications, and implant removals [6,20]. Concurrently, open reduction with internal fixation, particularly the modified Dunn osteotomy, has gained favor, while the use of in situ pinning and standalone epiphysiodesis as primary interventions has declined [4,5,13,23,24]. These shifts likely reflect a growing consensus that anatomic realignment optimizes long-term joint function and minimizes degenerative change [13,23,24].

Postoperative complications

FAI is among the most consequential long-term sequelae of SCFE, frequently precipitating early osteoarthritis and hastening the need for THA [4,5,13]. FAI arises from aberrant congruity between the femoral head and acetabulum and may develop even after anatomically mild slips have been corrected [4,5,13]. Three principal FAI morphologies are recognized, namely, Cam (inclusion), Pincer (impaction), and mixed, with the Cam type predominating in SCFE and the Pincer type portending the greatest severity [4,5,13].

For mild SCFE, in situ pinning remains the standard of care; however, adjunctive arthroscopic or open osteochondroplasty has demonstrated superior short- and mid-term outcomes in cohort studies by Leunig et al. and Chen et al. [12,25]. In moderate to severe slips, the modified Dunn procedure has been associated with a reduced incidence of subsequent FAI as this complication in patients who had undergone in situ fixation reported an incidence of 40% with considerably less in the modified Dunn group [4,5,9,13,23]. Alternative strategies such as acute closed reduction with pinning, urgent open "partial reduction" with stabilization, or open reduction and internal fixation have been proposed to mitigate impingement, though their long-term efficacy awaits validation through extended follow-up in specialized hip centers [4,5,13].

Initial management of FAI emphasizes conservative measures, which include but are not limited to activity modification, targeted strengthening, and anti-inflammatory pain control [4,5]. When nonoperative therapy fails, surgical options include femoral head-neck recontouring for mild to moderate impingement and proximal femoral osteotomies for severe deformities [4,5,12,13]. While combining realignment and recontouring procedures may offer theoretical benefits, differing postoperative protocols currently limit their simultaneous application [12,13]. These interventions aim to alleviate symptoms and forestall joint degeneration but are contraindicated once radiographic osteoarthritis is established; at that juncture, total hip replacement often becomes the definitive treatment [4,5,6,11].

A summary of the outcomes of each surgical intervention is highlighted in Table 3.

**Table 3:** Summarized comparison of surgical interventions with a descriptive emphasis on complications

Procedure	Description	Advantages	Disadvantages	Reference(s)
In situ fixation	Percutaneous insertion of cannulated screws across the physis without active correction of slippage	Minimally invasive. Slightly lower risk of avascular necrosis	Increased risk of total hip arthroplasty, especially with increased preoperative slip angle. The rate of impingement is 29%. The rate of avascular necrosis is 2.2%. The rate of subsequent corrective surgery is 4.4%. Lower screw threads and partially threaded screws resulted in worse outcomes	[26-28]
Open reduction and internal fixation	Open exposure of the hip joint followed by femoral head reduction and screw fixation	Better success rates with severe slips than with in situ fixation	Higher rate of avascular necrosis	[29]
Modified Dunn procedure	Surgical dislocation of the hip followed by the realignment of the epiphysis with preservation of the blood supply	Decreased need for corrective surgery. No conversion to total hip arthroplasty has been shown in multiple studies	11.1% rate of avascular necrosis in one study vs. 13% in another study; not statistically significant, with little risk of osteoarthritis described	[30-33]
Epiphysiodesis (physeal closure)	Fixation across the physis to halt growth	Minimally invasive as screws can be inserted percutaneously	5% risk of osteoarthritis. Typically recommended only with subsequent corrective osteotomy after physeal closure or if symptomatic impingement	[34-36]

Table 3 describes the complications associated with each of the practiced surgical interventions in managing SCFE. AVN is the most feared SCFE complication, resulting from the interruption of the femoral head's vascular supply and leading to collapse and joint degeneration [3,5]. Early diagnosis and treatment of unstable SCFE are critical to reduce AVN risk [5,10]. Loder demonstrated AVN rates of 14% in unstable slips treated by urgent reduction with internal fixation and decompressive arthrotomy, 5% following urgent open reduction, and 8% after the modified Dunn procedure [37]. Kohno et al.'s retrospective series found a significantly higher AVN rate (35%) in unstable slips managed with closed reduction versus 5.9% in those treated with in situ pinning, underscoring the risk of capsular pressure elevation during closed maneuvers.

When AVN occurs, progression to degenerative osteoarthritis is inevitable, often necessitating early THA [3,5,6,11,37,38].

Limb-length discrepancy is a frequent sequela of SCFE. Standard in situ stabilization with a cannulated screw transects the proximal femoral physis, arresting its growth while the contralateral physis continues to elongate, resulting in discrepancy [3,4,36].

Preserving femoral-neck growth and remodeling reduces the risk of limb-length inequality and secondary FAI. Consequently, "growth-preserving" fixation techniques have been advocated. These include the use of multiple smooth, non-threaded pins across the physis; the sliding, or "gliding", screw design; pin-and-sleeve constructs; and telescoping screws, all of which permit continued physeal growth while maintaining epiphyseal stability [3,4,36].

With regard to screw head impingement, postoperative groin pain soon after surgery often reflects screw-head impingement [3,4]. The risk increases when the screw is too long, for example, protruding beyond the subchondral bone, or when it is placed too anteriorly, where it catches on the acetabular rim [3,4]. Optimal placement requires the screw head to sit flush with the lateral cortex just below the vastus lateralis origin and the tip to be centered within the epiphysis on both anteroposterior (AP) and lateral views. Revision and repositioning of malpositioned screws reliably relieve symptoms [3,4].

Peri-implant femoral fracture can be explained through the presence of stress risers. Stress risers created by multiple screw entry holes or by aggressive bone removal during osteoplasty can precipitate peri-implant femoral fractures [4,39]. The risk is further elevated when implants are placed at or below the lesser trochanter (where cortical support is thin) or in the setting of postoperative osteomyelitis. Prevention strategies include limiting the number and diameter of cortical perforations, avoiding low trochanteric entry points, and preserving cortical integrity; treatment follows standard femoral fracture fixation principles, often requiring plate‐and‐screw constructs or intramedullary nailing [4,39].

Chondrolysis is a rapid degeneration and narrowing of the hip joint cartilage, which may occur after SCFE treatment [4,5]. Established risk factors include underlying autoimmune or endocrine disease, prolonged hip spica immobilization, subtrochanteric osteotomy before physeal closure, and, most commonly, intra-articular screw penetration [4,5,7]. Delayed diagnosis, obesity, and severe slips also increase risk. Management is primarily conservative, that is, protected weight-bearing, targeted muscle-strengthening exercises, and anti-inflammatory therapy, alongside the correction of any underlying metabolic or autoimmune condition [4,5,7].

Over time, smooth pins or unthreaded screws may loosen, migrate, or bend, resulting in recurrent hip pain and potential slip progression [3,4]. Migrated hardware can abrade cartilage, exacerbate FAI, or jeopardize the remaining blood supply, which heightens the risk of AVN. Routine postoperative radiographic surveillance is essential; symptomatic or malpositioned implants should be removed or exchanged promptly [3,4].

Excessive bone resection during osteochondroplasty or unrecognized preexisting acetabular dysplasia may compromise hip stability, leading to postoperative subluxation or dislocation. Over-contouring of the femoral head-neck junction reduces the weight-bearing surface and alters joint biomechanics. In cases of persistent instability, periacetabular osteotomy to reorient the acetabulum and improve femoral head coverage can restore stability and alleviate symptoms [4,5,12].

Discussion

SCFE is an increasingly prevalent hip pathology in adolescents that often carries lifelong sequelae [1-3]. There remains a significant opportunity to refine our understanding of outcome determinants and to standardize management across its heterogeneous presentations [3-5]. Contemporary anatomic reduction techniques, such as the modified Dunn osteotomy, are promising but technically demanding; their widespread adoption requires demonstrably low rates of AVN and other complications [13,23,24,32,33].

Long-term prognosis depends on age at presentation, slip severity, and chosen surgical intervention [3-5]. Early diagnosis, which can be achieved by thorough history-taking and careful clinical and radiographic evaluation, is critical to preserve hip function and delay, or potentially avoid, subsequent procedures. In situ fixation with pins or screws remains the mainstay for stable and minimally displaced (<30°) slips and may be employed prophylactically in the contralateral hip [1,4,5]. Even after successful stabilization, patients remain at risk for early osteoarthritis, chondrolysis, FAI, and AVN, underscoring the need for lifelong surveillance [3-6,11,34].

In moderate to severe slips managed with in situ fixation, posteromedial epiphyseal displacement places the metaphysis anterolaterally, predisposing to impingement against the anterior acetabular rim during hip flexion and eventual symptomatic FAI [4,5,13]. For unstable or severe SCFE, prompt surgical stabilization is imperative [4,5,13]. Although no approach is free of risk, consistent adherence to meticulous technique can mitigate impingement and other adverse outcomes [3,4,13]. Ongoing research must continue to optimize surgical strategies, minimize long-term morbidity, and establish evidence-based guidelines for both acute and prophylactic management [3-5,13,32].

## Conclusions

SCFE is a hip disorder affecting adolescents, often associated with obesity, puberty, and hormonal imbalances. It results from the femoral head slipping out of alignment, causing hip pain, limping, and limited mobility. Diagnosis typically involves X-rays, with MRI or CT scans used when needed. Treatment depends on the stability; a stable SCFE is typically managed with in situ fixation using a single screw, while an unstable SCFE may require open surgery, such as the modified Dunn procedure. With increasing adolescent obesity, SCFE incidence is rising, highlighting the need for optimized treatment strategies and long-term care. This review compared surgical methods to determine which yielded the fewest complications, especially long-term complications, such as AVN, deformities, and early arthritis. The Dunn procedure demonstrated superior radiographic and clinical outcomes, including lower AVN rates, improved function, and fewer reoperations compared to in situ pinning, especially in acute cases. As SCFE prevalence grows, continued research is essential to minimize complications and further improve the quality of life in patients with SCFE postoperatively. 
